# Metformin Attenuates Bone Cancer Pain by Reducing TRPV1 and ASIC3 Expression

**DOI:** 10.3389/fphar.2021.713944

**Published:** 2021-08-04

**Authors:** He-Ya Qian, Fang Zhou, Rui Wu, Xiao-Jun Cao, Tao Zhu, Hao-Dong Yuan, Ya-Nan Chen, Ping-An Zhang

**Affiliations:** ^1^ Department of Oncology, Affiliated Zhangjiagang Hospital of Soochow University, Zhangjiagang, China; ^2^ Center for Translational Medicine, Affiliated Zhangjiagang Hospital of Soochow University, Zhangjiagang, China; ^3^ Department of Laboratory, Affiliated Zhangjiagang Hospital of Soochow University, Zhangjiagang, China; ^4^ Center for Translational Pain Medicine, Institute of Neuroscience, Soochow University, Suzhou, China

**Keywords:** metformin, bone cancer pain, ASIC3, TRPV1, dorsal root ganglions

## Abstract

Bone cancer pain (BCP) is a common pathologic pain associated with destruction of bone and pathological reconstruction of nervous system. Current treatment strategies in clinical is inadequate and have unacceptable side effects due to the unclear pathology mechanism. In the present study, we showed that transplantation of Walker 256 cells aggravated mechanical allodynia of BCP rats (**p < 0.01 vs. Sham), and the expression of ASIC3 (Acid-sensitive ion channel 3) and TRPV1 was obviously enhanced in L4-6 dorsal root ganglions (DRGs) of BCP rats (**p < 0.01 vs. Sham). ASIC3 and TRPV1 was mainly expressed in CGRP and IB4 positive neurons of L4-6 DRGs. While, TRPV1 but not ASIC3 was markedly upregulated in L4-6 spinal dorsal horn (SDH) of BCP rats (**p < 0.01 vs. Sham). Importantly, intrathecal injection of CPZ (a TRPV1 inhibitor) or Amiloride (an ASICs antagonist) markedly increased the paw withdraw threshold (PWT) of BCP rats response to Von Frey filaments (**p < 0.01 vs. BCP + NS). What’s more, intraperitoneally injection of Metformin or Vinorelbine markedly elevated the PWT of BCP rats, but reduced the expression of TRPV1 and ASIC3 in L4-6 DRGs and decreased the TRPV1 expression in SDH (*p < 0.05, **p < 0.01 vs. BCP + NS). Collectively, these results suggest an effective analgesic effect of Metformin on mechanical allodynia of BCP rats, which may be mediated by the downregulation of ASIC3 and TRPV1.

## Introduction

Pain is a common symptom in cancer patients (Sheng and Zhang, 2020). Previous studies have shown that 85% of patients with lung, breast and prostate cancer appear bone metastasis, and about one-third of them was accompanied with bone cancer pain (BCP) symptoms (Okui et al., 2021). Bone cancer pain (BCP) is a unique pain that includes features of nociceptive, neuropathic, and inflammatory pain (de Clauser et al., 2020). BCP can produce an excruciating pain, but current treatments strategies may be inadequate or have unacceptable side effects (Sindhi and Erdek, 2019; Yu et al., 2020; Chen et al., 2021). Therefore, it is urgent to explore the new mechanisms and develop novel therapeutic targets to relieve BCP. Metformin, serving as an antidiabetic drug (Yu et al., 2019), is recently revealed to exist anti-cancer properties by regulation of cancer-relevant signaling pathways (Cao et al., 2016). Additionally, Metformin was proved to exert analgesic effect in neuropathic pain (Hacimuftuoglu et al., 2020), inflammatory pain (Russe et al., 2013) and visceral pain (Nozu et al., 2019). However, the effects and mechanism of Metformin in chronic pain induced by cancer bone metastasis remains unknown.

Metabolize of bone cancer cell could produce acidic environment (George et al., 2020). It was reported that the mechanisms of bone cancer pain include tumor cells-induced nerve injury, central sensitization, tumor cells themselves, osteoclast-mediated osteolysis as well as acidic environment of bone tissue (Nagae et al., 2007; Mantyh, 2014; Romero-Morelos et al., 2021). The Acid-sensitive ion channels (ASICs) is one of the most sensitive ion channels in detecting changes in pH (Mir and Jha, 2021). ASIC3 has the highest content in dorsal root ganglions (DRGs) and is sensitive to extracellular acidification. Additionally, transient receptor potential ion channel (TRPV1), a nonselective cation channel, can also be activated by H^+^ (Elokely et al., 2016; Wu et al., 2019; Rosenberger et al., 2020). TRPV1 is highly enriched in dorsal root ganglions (DRGs), and its involvement in diverse pain states has been well documented (Sondermann et al., 2019). TRPV1 represents a major player in pathological pain arising from inflamed and injured tissues, i.e., inflammatory pain and cancer pain (Joseph et al., 2019). However, whether ASIC3/TRPV1 is involved in the analgesic effect of Metformin on bone cancer pain remains unclear.

In the present study, we revealed that Metformin exerted therapeutic effects on bone cancer pain. Furthermore, we proved that the mechanism underlying analgesic effect of Metformin was mediated by regulation of TRPV1 and ASIC3 expression.

## Materials and Methods

### Animals

Female Sprague-Dawley (SD) rats (180–220 g) were housed in a temperature- (24 ± 1°C) and light-controlled (12/12-h light-dark cycle) room with free access to food and water. All experimental procedures were approved by the Laboratory Animal Center of Soochow University (Suzhou, Jiangsu, P.R. China).

### Generation of the Bone Cancer Pain Model

The BCP model was established according to the method previously reported (Zhou et al., 2015; Hu et al., 2019; Shenoy et al., 2019; Zhang et al., 2020). In detail, the young rat was intraperitoneally injected with 2 × 10^7^ Walker 256 cells. One week later, the ascites fluids were extracted and the cells were collected. After that, the Walker 256 cells were counted and diluted to a final concentration of 1 × 10^8^ cells/ml. The Walker 256 cells (4 × 10^5^) was then slowly injected into the tibia cavity using a microinjection syringe with a 23-gauge needle. In the Sham group, the same volume of normal saline solution was injected into the tibial medullary cavity of rats. In the naïve group, the rats did not suffer any treatment.

### Evaluation of Mechanical Allodynia

The “up and down” method was used to evaluate mechanical allodynia (Zhou et al., 2015) and the 50% paw withdrawal threshold (PWT) was determined as previously described methods. In detail, the rats were placed in a transparent box and allowed to acclimate for 30 min. The experiments were performed in a double-blinded manner. A series of standard Von Frey filaments (VFFs) (0.6, 1.0, 1.4, 2.0, 4.0, 6.0, 8.0, 10.0, 15.0 and 26.0 g) were vertically advanced to the plantar surface of the hind paw using sufficient force until the filament bent. The trial began with the filament possessing a buckling weight of 8.0 g. Withdrawal of the hind paw was regarded as a positive response, and a weaker stimulus was presented. In the absence of response, a stronger stimulus was applied.

### Western Blotting

The expressions of TRPV1 and ASIC3 in the dorsal root ganglia (DRGs) and spinal dorsal horn (SDH) were determined using western blotting. After the rats were sacrificed, the L4–L6 spinal dorsal horn and DRGs were collected and lysed. The lysates were centrifuged at 12,000 g at 4°C. The supernatant was collected and the total protein concentration was qualified using a bicinchoninic acid (BCA) protein assay kit (Thermo Scientific, MA, United States). Equal amounts of proteins were separated by 10% polyacrylamide gels (Bio-Rad, CA, United States). Then the proteins were transferred to polyvinyli denedifluoride membranes. The membranes were blocked in a 5% non-fat milk for 2 h at room temperature and incubated with the primary antibodies [anti-TRPV1 (Genetex, United States), anti-ASIC3 (Genetex, United States), and anti-GAPDH (Abcam, Cambridge, United Kingdom)] at 4°C overnight. After washing with TBST (0.5% Tween-20), the membranes were then incubated with peroxidase-conjugated secondary antibodies for 2 h at room temperature. An imaging system (Bio-Rad, CA, United States) was used to examine chemiluminescence. Expression of protein was normalized to that of GAPDH.

### Real-Time Quantitative Polymerase Chain Reaction

Total RNA was extracted from the L4-L6 DRGs and SDH using Trizol (Invitrogen, CA) method. cDNA was synthesized from total RNA using an Omniscript RT kit 50 (Qiagen, Valencia, CA) according to the manufacturer’s instructions. The primers for TRPV1, ASIC3, and GAPDH (internal control) were used. The primer sequences are showed in Table 1. To evaluate the expressions of genes, 2^-△△Ct^ values were calculated. The mRNA expression values of TRPV1 and ASIC3 were normalized to that of GAPDH.

**TABLE 1:** Primers used for real-time PCR.

Name	Forward primer	Reverse primer
TRPV1	GCT​CTG​GTG​CTT​GGC​TAT​GA	GGG​TCG​ACC​TGA​TAC​TTG​GC
ASIC3	GTG​GTG​CTG​GCA​ACG​GAC​TG	GGC​TCA​TCC​TGG​CTG​TGA​ATC​TG
GAPDH	AAG​GTG​GTG​AAG​CAG​GCG​GC	GAG​CAA​TGC​CAG​CCC​CAG​CA

### Immunofluorescence Assay

The Immunofluorescence assay was conducted as described in previous report (Zhou et al., 2015). In brief, the rats were anesthetized and transcardially perfused with a saline solution followed by 4% paraformaldehyde (Sinopharm Chemical Reagent Co. Ltd., Shanghai, P.R. China). The L4-6 DRGs were then removed and fixed in paraformaldehyde and dehydrated in 10, 20 and 30% sucrose (Sinopharm Chemical Reagent Co. Ltd.) in succession until sinking. The DRGs were cut at 14 μm thickness using freezing microtome (Leica, Wetzlar, Germany). The sections were incubated with blocking solution, and followed by the primary antibodies [anti-TRPV1 (Genetex, United States), anti-ASIC3 (Genetex, United States), anti-NeuN (Merck Millipore, Germany), anti-GS (Abcam, Cambridge, United Kingdom), anti-calcitonin gene-related peptide (CGRP) (Abcam, Cambridge, United Kingdom), anti-neurofilament (NF)-200 (Abcam, Cambridge, United Kingdom), anti-isolectin B4 (IB4) (Sigma, St. Louis, MO)] at 4°C overnight. After washing with PBS, the secondary antibodies labeled Alexa Fluor 488 and 555 (Molecular Probes, NY, United States) were incubated at room temperature for 1 h. The slides were observed under a fluorescence microscope, and the images were trimmed with AxioVision (Jena, Germany). Negative controls were performed by omitting the primary antibody.

### Drug Administration

Capsazepine (HY-15640) was purchased from Medchemexpress (Monmouth Junction, NJ, United States). Amiloride (T0175) and Vinorelbine (T6213) were purchased from TargetMol (Wellesley Hills, MA, United States). Metformin was purchased from Sigma-Aldrich (St. Louis, MO, United States). After acclimatization for 1 week, the rats were injected with Walker 256 cells. After 2 weeks, twenty-four rats were divided into four groups. In group 1, rats were received an intrathecal injection of amiloride (0.1 mg/10 μL), In group 2, rats were received a subcutaneous injection of 15 mg/kg capsazepine (CPZ). In group 3, rats were received an intraperitoneal injection of 3 mg/kg Vinorelbine. In group 4, rats were received an intraperitoneal injection of 200 mg/kg Metformin (Ge et al., 2018; Hacimuftuoglu et al., 2020). The PWT was recorded at 0.5, 1, 2, 4, and 8 h, and 2, 5, 7, 14, and 21 days after the administrations of drugs. To evaluate the long-term effects of Metformin, the rats were received Metformin injections once a day for five consecutive days.

### Data Analysis

All data were analyzed using SPSS 17.0 (SPSS, Chicago, IL, United States) and Origin Pro 8 (OriginLab, Northampton, MA). All Data were showed as Mean ± SEM. Statistical analyses were performed by two-way analysis of variance (ANOVA) with multiple comparisons. Normality was verified for all data before analyses. *p* < 0.05 was considered as a statistical significance.

## Results

### Transplantation of Walker 256 Cells Increased the Expression of Transient Receptor Potential Ion Channel and ASIC3

**FIGURE 1:**
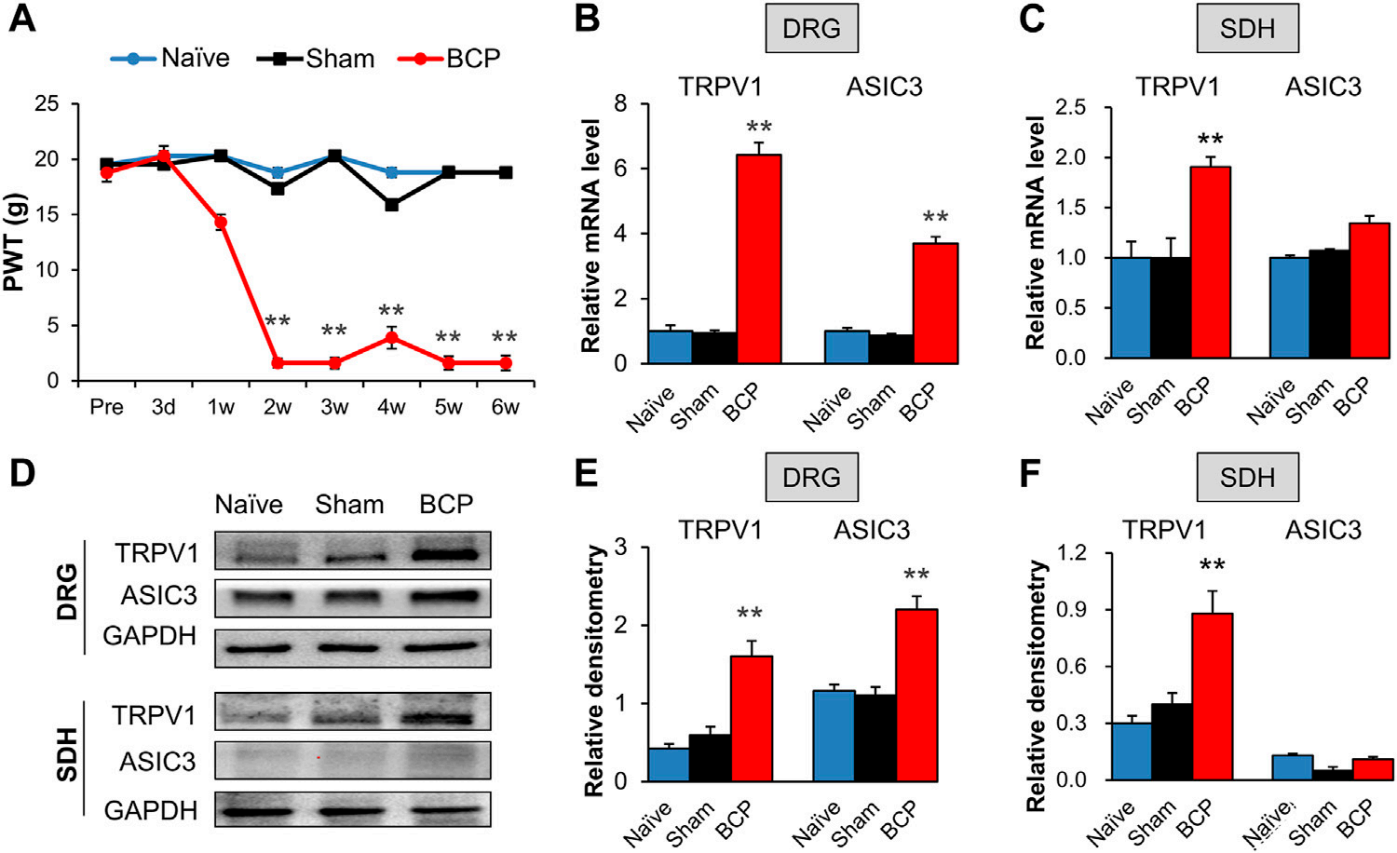
The expression of TRPV1 and ASIC3 was increased in BCP rats. **(A)** PWT was significantly reduced 2 weeks after tumor cell compared with the Naïve group (SD healthy female rats with not any treatment) and Sham group (normal saline injection) (**p < 0.01 vs. Sham, two-way repeated measures ANOVA followed by Tukey’s post hoc test). **(B)** Quantification of QPCR assays showing significant up-regulation of TRPV1 and ASIC3 mRNA expression in L4-6 DRGs of BCP rats at 2 weeks after transplantation compared with Sham rats (**p < 0.01 vs. Sham, two-way repeated measures ANOVA followed by Tukey’s post hoc test). **(C)** Quantification of QPCR assays showing significant up-regulation of TRPV1 mRNA expression in L4-6 spinal dorsal horn (SDH) of BCP rats at 2 weeks after transplantation compared with Sham rats (**p < 0.01 vs. Sham, two-way repeated measures ANOVA followed by Tukey’s post hoc test). While mRNA expression of ASIC3 in L4-6 spinal dorsal horn (SDH) of BCP rats at 2 weeks after transplantation was not altered compared with Sham rats (*p* > 0.05 vs. Sham, two-way repeated measures ANOVA followed by Tukey’s post hoc test). **(D)** Immunoblot showed the protein level of TRPV1 and ASIC3 in DRGs and SDH of Naïve, Sham and BCP rats. **(E)** The protein expression of TRPV1 and ASIC3 in the ipsilateral L4-6 DRGs of BCP rats was significantly increased compared with Sham rats at 2 weeks after transplantation (**p < 0.01 vs. Sham, two-way repeated measures ANOVA followed by Tukey’s post hoc test). **(F)** Protein expression of TRPV1 in the ipsilateral L4-6 SDH of BCP rats was significantly increased compared with Sham rats at 2 weeks after transplantation (**p < 0.01 vs. Sham, two-way repeated measures ANOVA followed by Tukey’s post hoc test). While protein ASIC3 expression was not altered. (*p* > 0.05 vs. Sham, two-way repeated measures ANOVA followed by Tukey’s post hoc test).

We evaluated the PWT of rats before transplantation, and no signs of mechanical allodynia were observed in each group (Figure 1A, Pre). The result demonstrated that the PWT of BCP rats showed a significant decrease on the 2nd week after transplantation of Walker 256 cells when compared with that of Sham group (Figure 1A, **p < 0.01 vs. Sham), and the decrease lasted to at least 6 weeks (Figure 1A, **p < 0.01 vs. Sham). The PWT of rats showed no difference between naïve and Sham group. The result indicates that transplantation of Walker 256 cells aggravated mechanical allodynia of rats.

The mRNA and protein levels of TRPV1 and ASIC3 in the ipsilateral L4-6 DRGs and spinal dorsal horn (SDH) were assessed to determine their role in mechanical allodynia of BCP rats. QPCR results showed that the mRNA levels of TRPV1 and ASIC3 were significantly increased in the ipsilateral DRGs of BCP rats compared with Sham ones (Figure 1B, **p < 0.01 vs. Sham). Additionally, the mRNA level of TRPV1 but not ASIC3 was markedly increased in the ipsilateral SDH of BCP rats (Figure 1C, **p < 0.01 vs. Sham). The up-regulation was further confirmed by western blotting (Figure 1D). The results showed that the protein levels of TRPV1 and ASIC3 were obviously increased in the ipsilateral DRGs (Figure 1E, **p < 0.01 vs. Sham). And, the protein level of TRPV1 but not ASIC3 was markedly increased in the ipsilateral SDH of BCP rats compared with Sham ones (Figure 1F, **p < 0.01 vs. Sham). These results indicate that the expression of TRPV1 and ASIC3 was enhanced in BCP rats.

### Transient Receptor Potential Ion Channel and ASIC3 Were Mainly Expressed in the Dorsal Root Ganglions Neurons

**FIGURE 2:**
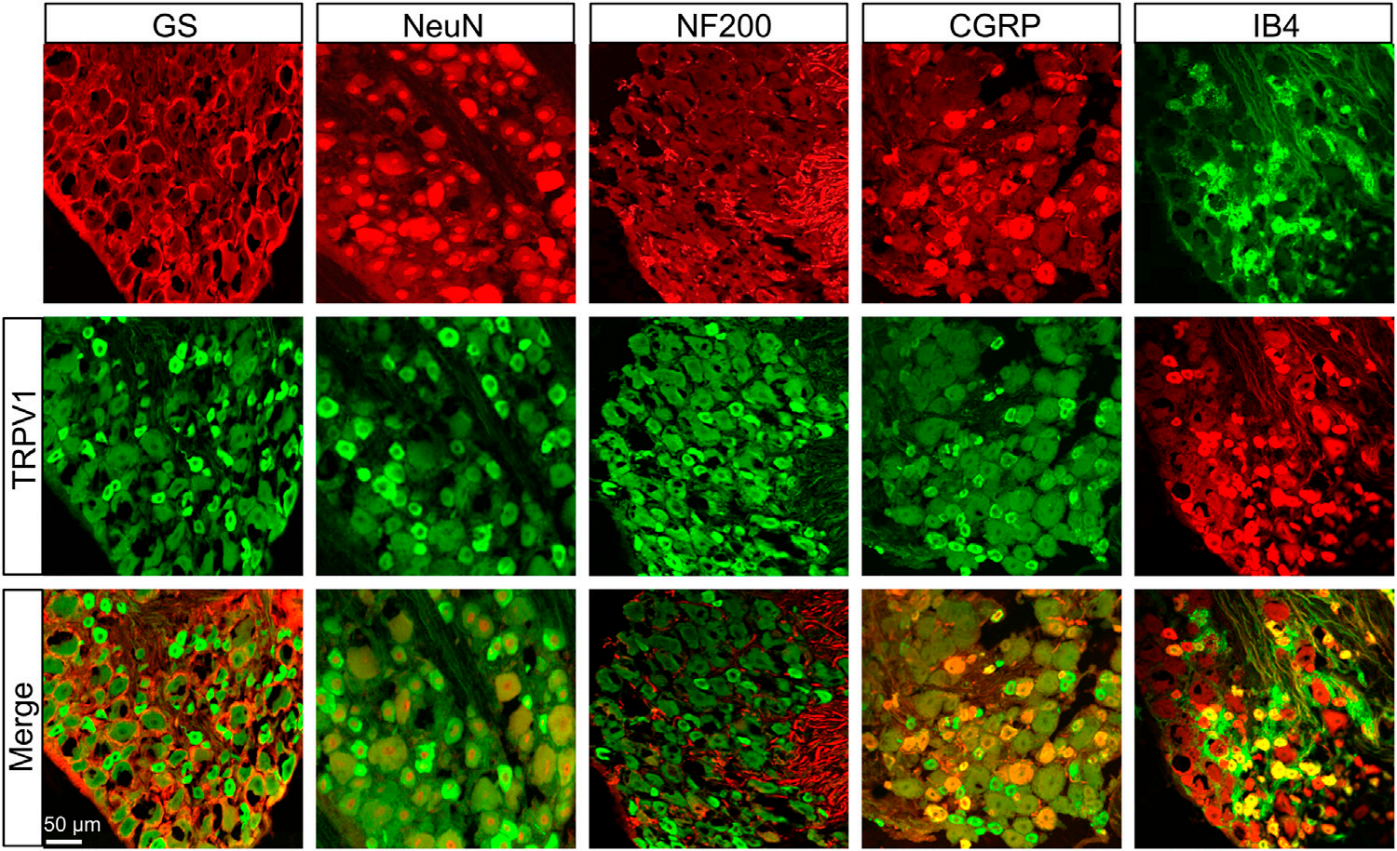
Immunofluorescence assay of TRPV1 in L4-6 DRGs. TRPV1-positive DRG cells (middle row) were co-labeled as neuron-positive (upper left second, red) but not with GS-positive cells (upper left first, red). TRPV1 was co-expressed in IB4-positive (upper right first, green) and CGRP-positive (upper right second, red) DRG neurons. TRPV1 was a few expressed in NF-200-positive (upper middle, red) DRG neurons. Merges of TRPV1 with GS, NeuN, CGRP, NF-200, and IB4 are shown in the lower row. Scale bar, 50 μm. Quantification showing that TRPV1 was mainly located in small and medium sensory neurons labeled with IB4 and CGRP but a few with NF-200.

**FIGURE 3:**
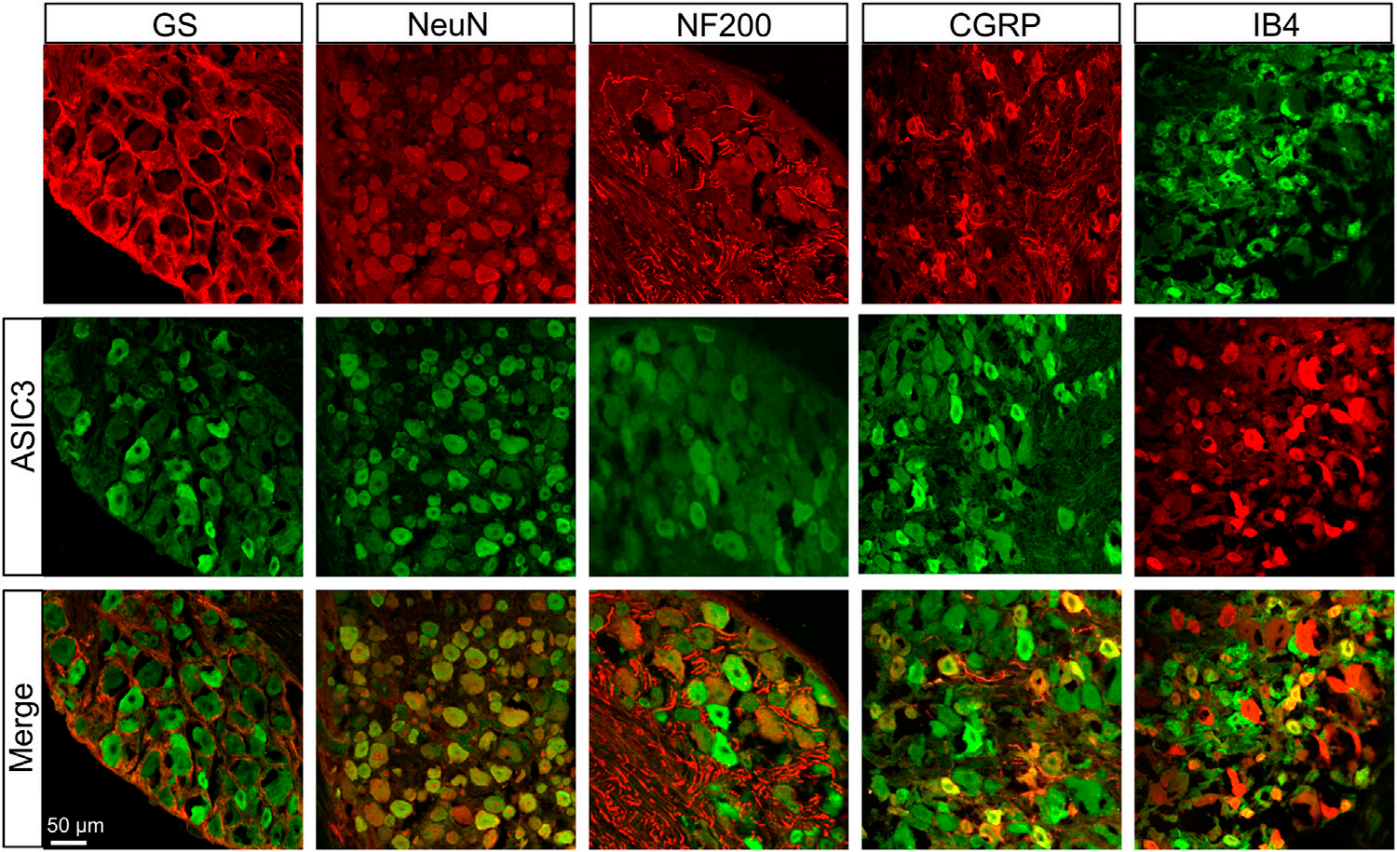
Immunofluorescence assay of ASIC3 in L4-6 DRGs. ASIC3-positive DRG cells (middle row) were co-labeled as neuron-positive (upper left second, red) but not with GS-positive cells (upper left first, red). ASIC3 was co-expressed in IB4-positive (upper right first, green) and CGRP-positive (upper right second, red) DRG neurons. ASIC3 was a few expressed in NF-200-positive (upper middle, red) DRG neurons. Merges of ASIC3 with GS, NeuN, CGRP, NF-200, and IB4 are shown in the lower row. Scale bar, 50 μm. Quantification showing that ASIC3 was mainly located in small and medium sensory neurons labeled with IB4 and CGRP but a few with NF-200.

We further investigated the distributions of TRPV1 and ASIC3 in the DRG using immunofluorescence assay. As shown in Figure 2, TRPV1 was primarily expressed in neurons (labeled by NeuN) but not satellite glial cells (labeled by GS). Furthermore, we co-stained TRPV1 with NF-200 (a marker of large neurons), isolectin B4 (IB4, a marker of non-peptidergic small and medium neurons), and calcitonin gene-related peptide (CGRP, a marker of small and medium peptidergic neurons) by immunofluorescence and the result showed that TRPV1 was mainly expressed in small and medium neurons labeled with IB4 and CGRP. Additionally, we also investigated the distributions of ASIC3 in DRGs. Immunofluorescence assay showed that ASIC3 was mainly co-localized with NeuN, CGRP and IB4, but not with GS and a little with NF200 (Figure 3), indicating ASIC3 was mainly expressed in small and medium sensory neurons of L4-6 DRGs.

### Injection of Capsazepine or Amiloride Attenuated Mechanical Allodynia of Bone Cancer Pain Rats

**FIGURE 4:**
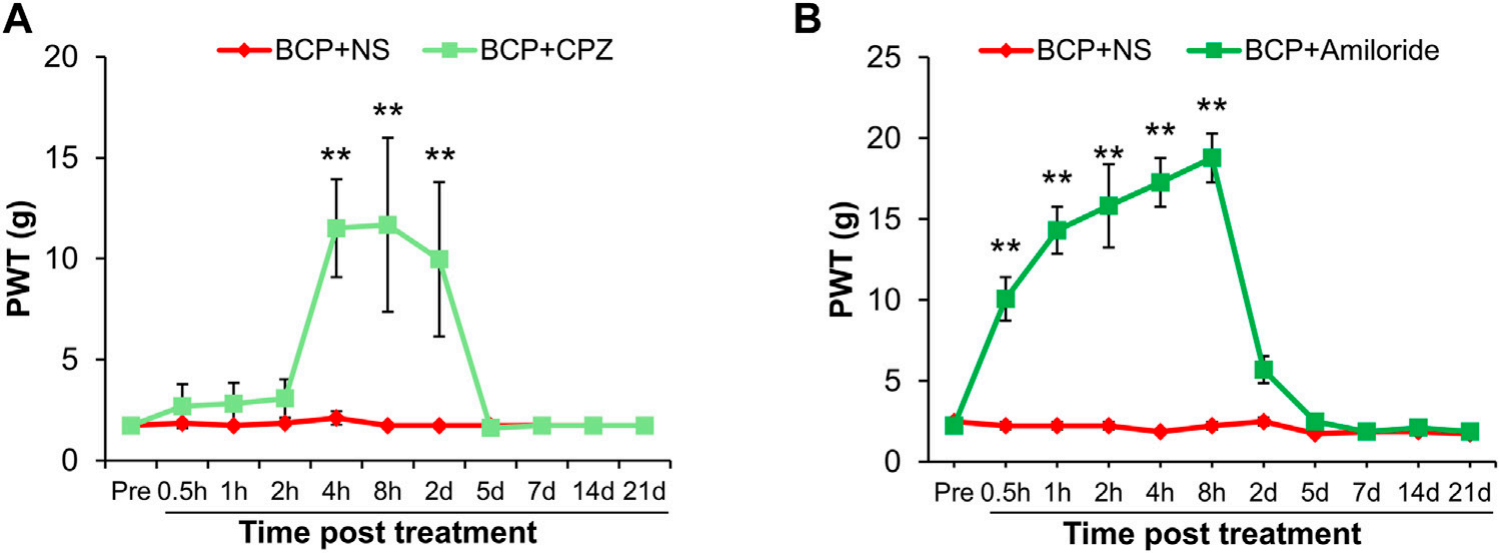
The PWT of BCP rats was increased after Capsazepine (CPZ) or Amiloride injection. **(A)** The PWT of BCP rats (2 weeks after transplantation) subcutaneous injected with TRPV1 inhibitor (CPZ, 15 mg/kg) were significantly increased than in age-matched BCP rats injected with same volume of NS from 4 h to 2 days (**p < 0.01 vs. BCP + NS, two-way repeated measures ANOVA followed by Sidak’s post hoc test). **(B)** The PWT of BCP rats (2 weeks after transplantation) intrathecal injected with ASIC3 inhibitor (Amiloride, 0.1 mg/10 μL) were significantly increased than in age-matched BCP rats injected with the same volume of NS from 0.5 to 8 h (**p < 0.01 vs. BCP + NS, two-way repeated measures ANOVA followed by Sidak’s post hoc test).

To investigate the role of TRPV1 and ASIC3 in the mechanical allodynia, their antagonist CPZ and Amiloride was separately intrathecal injected in BCP rats. As shown in Figure 4A, the PWT of BCP rats were markedly increased after CPZ injection from 4 h to 2 days compared with normal saline (NS) treated BCP rats (**p < 0.01 vs. BCP + NS). Similarly, the BCP rats treated with Amiloride showed an increase of PWT from 0.5 to 8 h (Figure 4B, **p < 0.01 vs. BCP + NS). These data suggest that up-regulated TRPV1 and ASIC3 contribute to the mechanical allodynia of BCP rats.

### Metformin and Vinorelbine Attenuated Mechanical Allodynia of Bone Cancer Pain Rats

**FIGURE 5:**
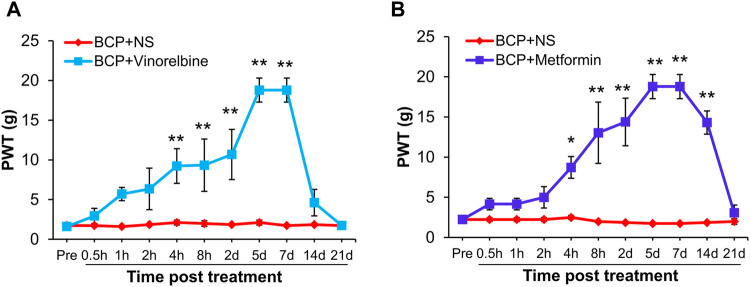
Metformin or Vinorelbine treatment attenuated the mechanical allodynia of BCP rats. **(A)** The PWT of BCP rats (2 weeks after transplantation) treated with Vinorelbine (intraperitoneal injection, 3 mg/kg) was significantly increased than in age-matched BCP rats injected with same volume of NS from 4 h to 7 days (**p < 0.01 vs. BCP + NS, two-way repeated measures ANOVA followed by Sidak’s post hoc test). **(B)** The PWT of BCP rats (2 weeks after transplantation) treated with Metformin (intraperitoneal injection, 200 mg/kg) was significantly increased than in age-matched BCP rats injected with same volume of NS from 4 h to 14 days (*p < 0.5, **p < 0.01 vs. BCP + NS, two-way repeated measures ANOVA followed by Sidak’s post hoc test).

Next, we evaluated the effects of Metformin and Vinorelbine (a clinical chemotherapeutic drugs) on mechanical allodynia of BCP rats. After transplanted cancer cells for 2 weeks, the rats were treated with Metformin or Vinorelbine separately. As shown in Figure 5A, the PWT of BCP rats treated with Vinorelbine was continuously increased from 4 h to 7 days, and was most obvious from 5 to 7 days (**p < 0.01 vs. BCP + NS). Similarly, in the Metformin group, the PWT of BCP rats increased from 4 h to 14 days after treatment of Metformin, and the effect lasted to at least 14 days (Figure 5B, *p < 0.05, **p < 0.01 vs. BCP + NS).

### Metformin or Vinorelbine Injection Reduced ASIC3 and Transient Receptor Potential Ion Channel Expression

**FIGURE 6:**
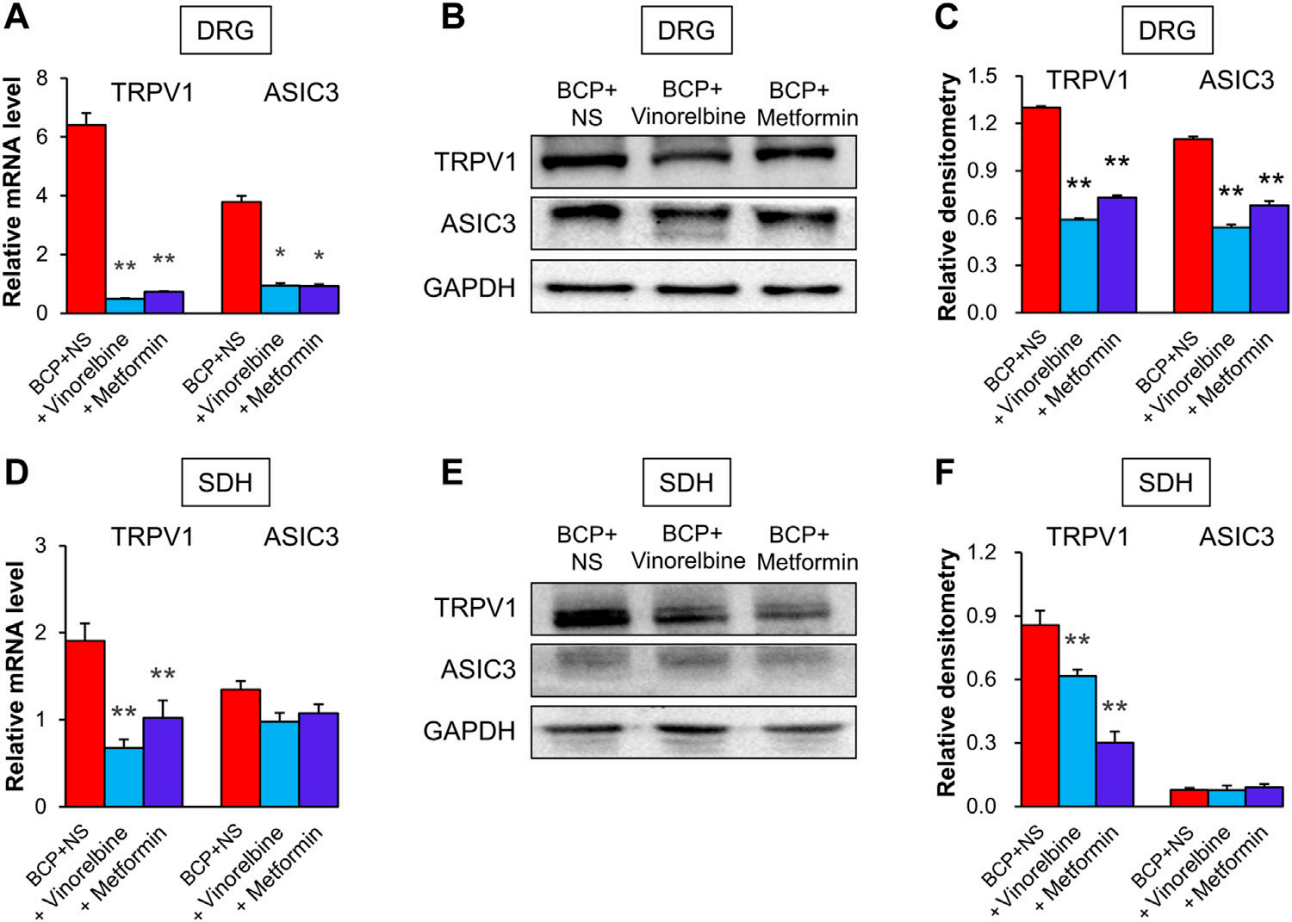
Metformin and Vinorelbine decreased the expressions of TRPV1 and ASIC3. **(A)** Quantification of qPCR assays showed that the mRNA level of TRPV1 and ASIC3 were decreased in the ipsilateral DRGs of BCP rats treated with Vinorelbine or Metformin when compared with BCP + NS rats (*p < 0.5, **p < 0.01 vs. BCP + NS, two-way repeated measures ANOVA followed by Tukey’s post hoc test). **(B)** Immunoblot showed the protein level of TRPV1 and ASIC3 in rat DRGs of BCP + NS, BCP + Vinorelbine, BCP + Metformin group. **(C)** The expression of TRPV1 and ASIC3 at the protein level in the ipsilateral DRGs of BCP rats treated with Vinorelbine or Metformin was greatly decreased when compared with BCP + NS rats (**p < 0.01 vs. BCP + NS, two-way repeated measures ANOVA followed by Tukey’s post hoc test). **(D)** QPCR assays showed that the mRNA level of TRPV1 was decreased in the ipsilateral SDH of BCP rats treated with Vinorelbine or Metformin when compared with a BCP + NS rats (**p < 0.01 vs. BCP + NS, two-way repeated measures ANOVA followed by Tukey’s post hoc test). While the mRNA level of ASIC3 was not changed (*p* > 0.05 vs. BCP + NS, two-way repeated measures ANOVA followed by Tukey’s post hoc test). **(E)** Immunoblot showed the protein level of TRPV1 and ASIC3 in rat SDH of BCP + NS, BCP + Vinorelbine, BCP + Metformin group. **(F)** The expression of TRPV1 at the protein level was decreased in the ipsilateral SDH of BCP rats treated with Vinorelbine or Metformin when compared with BCP + NS rats (**p < 0.01 vs. BCP + NS, two-way repeated measures ANOVA followed by Tukey’s post hoc test). While the ASIC3 expression was not changed (*p* > 0.05 vs. BCP + NS, two-way repeated measures ANOVA followed by Tukey’s post hoc test).

To evaluate the effects of Vinorelbine and Metformin on the expression of TRPV1 and ASIC3, qPCR and Western blotting was performed. The results showed that treatment of Vinorelbine or Metformin significantly inhibited the mRNA levels of TRPV1 and ASIC3 in the ipsilateral DRG when compared with those in the BCP + NS group (Figure 6A, *p < 0.05, **p < 0.01 vs. BCP + NS). Additionally, treatment of Metformin or Vinorelbine significantly reduced the protein levels of TRPV1 and ASIC3 in the ipsilateral DRG when compared with those in the BCP + NS group (Figures 6B,C, **p < 0.01 vs. BCP + NS). Additionally, the mRNA level of TRPV1 but not ASIC3 was obviously reduced in the ipsilateral SDH of BCP rats treated with Metformin or Vinorelbine (Figure 6D, **p < 0.01 vs. BCP + NS). Furthermore, the protein level of TRPV1 but not ASIC3 was remarkedly decreased in the ipsilateral SDH of BCP rats treated with Metformin or Vinorelbine (Figures 6E,F, **p < 0.01 vs. BCP + NS). The results demonstrate that treatment of Metformin or Vinorelbine decreased the expression of ASIC3 and TRPV1, suggesting a possible involvement of ASIC3 and TRPV1 in Metformin/Vinorelbine-mediated analgesic effect on BCP rats.

## Discussion

An important finding in the present study is to reveal the therapeutic potential of Metformin on bone cancer pain (BCP). Although numerous previous studies have demonstrated that Metformin can be used in the treatment of many types of cancers (Al Hassan et al., 2018; Roshan et al., 2019; Xue et al., 2019), the effects of Metformin on bone cancer pain are still unclear. In the present study, we found that the PWT of mechanical allodynia in BCP rats continued to increase for at least 14 days after treatment of Metformin, indicating that Metformin therapy could attenuate the bone cancer pain symptoms and the analgesic effect of Metformin was much better than Vinorelbine (last for 7 days). Notably, we revealed that the underlying analgesic mechanisms of Metformin on bone cancer pain are in part *via* reducing the expression of TRPV1 and ASIC3.

Firstly, we successfully established a rat model of BCP with the decreased PWT following the up-regulation of TRPV1 in both DRGs and spinal dorsal horn (SDH). Additionally, inhibition of TRPV1 by CPZ was able to ameliorate PWT of BCP rats, and a significant reduction in mechanical allodynia was observed from 8 h to 5 days after CPZ injection. This is consistent with the view that TRPV1 blockage or deletion is proved to be an effective method to attenuate chronic bone pain state (Heo et al., 2017; Hanaka et al., 2018). Importantly, Metformin application could significantly reduce the up-regulated TRPV1 in both DRG and SDH of BCP rats as well as Vinorelbine treatment, suggesting that TRPV1 might mediate the analgesic effect of Metformin and Vinorelbine on BCP. However, the regulation procedure of TRPV1 expression by Metformin and Vinorelbine was unknown and need to be investigated in the future research.

ASIC3 is another important protein that we proved here to contribute to the analgesic effect of Metformin. The increase of ASIC3 in the DRG neuron have been reported in a rat model of cancer-induced pain model (Qiu et al., 2014), and Wu et al. (2004) suggested that ASIC3 expressed little or no expression in rat spinal dorsal horn. Consistent with this, our data showed an increased ASIC3 in DRG neurons but the expression of ASIC3 was not changed in SDH, and the inhibitor Amiloride significantly elevated the PWT of BCP rats. Although inhibitors for ASIC3 showed effectiveness in BCP model, we noticed that the analgesic effect of these inhibitors only lasted for 8 h. Therefore, it is necessary to discover specific agents that are effective with longer-last effects for bone cancer pain such as Metformin. Furthermore, here we provided some evidences to confirm the involvement of ASIC3 in the analgesic effect of Metformin. Metformin treatment obviously reversed the upregulation of ASIC3 in DRGs. However, the regulation mechanism of Metformin in ASIC3 expression was not clear.

## Conclusion

Taken together, in the present study, we revealed that Metformin exerted therapeutic effects on bone cancer pain in a rat model. Treatment of Metformin reduced the mechanical allodynia of BCP rats for 14 days (better than Vinorelbine). Additionally, the mechanisms underlying analgesic effect of Metformin and Vinorelbine on BCP are in part *via* the downregulation of TRPV1 and ASIC3.

## Data Availability

The raw data supporting the conclusion of this article will be made available by the authors, without undue reservation.
